# Inhibition of pyruvate dehydrogenase kinase influence microbiota and metabolomic profile in pancreatic cancer xenograft mice

**DOI:** 10.1186/s13104-020-05384-9

**Published:** 2020-11-18

**Authors:** Kaarel Adamberg, Raivo Vilu, Valerio Pazienza

**Affiliations:** 1 Department of Food Processing, Tallinn University of Technology, Tallin, Estonia; 2 Department of Chemistry, Tallinn University of Technology Estonia, Tallin, Estonia; 3 Competence Center of Food and Fermentation Technologies, Tallin, Estonia; 4 Gastroenterology Unit I.R.C.C.S. “Casa Sollievo Della Sofferenza” Hospital, viale dei Cappuccini n.1, 71013 San Giovanni Rotondo, FG Italy

**Keywords:** Pancreatic cancer, Microbiota, Metabolomic

## Abstract

**Objective:**

Despite recent advances in treatment options, pancreatic cancer remains the most deadly major cancer. Targeting metabolism represents an emerging anti-cancer strategy.

**Results:**

Metagenomic 16S analysis was employed to explore the effect of Dichloroacetate (DCA) on the composition of the fecal microbiota and metabolomic profile was assessed on in vivo pancreatic cancer mouse xenograft model. Pancreatic cancer xenograft mice displayed a shift of microbiota’ profile as compared to control mice without DCA treatment and a significant decrease of the purine bases inosine xanthine together with their metabolically-related compound hypoxanthine were observed in the DCA treated group as compared to the control group. Two aminoacids methionine and aspartic acid resulted decreased and increased respectively. DCA affects tumor environment and studies are needed in order to understand whether DCA supplementation could be supportive as synergistic approach to enhance the efficacy of existing cancer treatments in pancreatic cancer patients.

## Introduction

Pancreatic cancer (PC) is one of the most aggressive and highly lethal tumor ranked as the fourth leading cause of cancer-related deaths worldwide and it is expected to become the second leading cause of cancer death in the next decade. Despite recent advances in treatment options, pancreatic cancer has the worst survival rate of any organ site, with a median survival of less than six months and a 5-year survival rate of less than 5% [1]. Due to the lack of early symptoms and biomarkers that can predict the onset of PC, the latter is often not diagnosed until it is advanced and therefore is habitually referred to as a “silent killer”. The available therapeutic strategies based on surgery and conventional chemotherapy are still essentially insufficient. Since metabolic reprogramming contributes to tumor development, targeting identified metabolic targets might represent a promising effective approach to treat pancreatic cancer, overcome chemoresistance and ameliorate patient’s prognosis and survival [2].

It is well known that tumor cells rely on glucose and glycolysis as a major source of energy [3] hence there is also a growing interest in fasting or calories restriction to prevent or treat cancer [4, 5]. It was previously demonstrated that rewiring carbohydrates metabolism differentially affects survival of pancreatic cancer cell lines [3] or retard pancreatic cancer tumor growth in pancreatic cancer in vivo model [6]. Pyruvate dehydrogenase kinase (PDK) is one of the key enzyme which increases the flux of pyruvate into the mitochondria, promoting glucose oxidation over glycolysis through the inactivation of pyruvate dehydrogenase (PDH) hence PDK1 inhibition by the generic drug orally available small molecule dichloroacetate (DCA) increasing pyruvate oxidation and oxidative stress contributes to cell death could represent a potential metabolic-targeting therapy for cancer [7]. We recently demonstrated that DCA-treated PANC-1 and BXPC-3 cells showed a marked decrease in cell proliferation and migration combined with an enhanced ROS production resulting in a mitigation of tumor growth in pancreatic cancer xenografted mice [8].

Being DCA an effective modulator of glucose metabolism, in this manuspcript we assessed the effect of DCA on microbiota and metabolomic profile on the above mentioned in vivo model of pancreatic cancer xenografted mice.

## Main text

### Materials and methods

#### Animal studies

All mice were housed and cared for in a GenScript facility accredited by the Association for Assessment and Accreditation of Laboratory Animal Care International (AAALAC International). A total number of 5 × 10^6^ BxPC‐3‐luc cancer cells per mouse was suspended in 0.1 mL of PBS/ matrigel mixture (1:1) and then injected into 5–6 weeks old female Nu/Nu mice (right flank) maintained in a specific pathogen-free (SPF) environment throughout the experiments. BxPC‐3‐luc tumor‐bearing nude mice were randomly assigned into 2 groups (6 mice/ group) when tumor size reached an average volume of 100 mm^3^. Group 1 (Normal saline, i.p, qw), group 2 (DCA, 100 mg/kg/day free drinking). Animals were humanely euthanized by CO2.

### Microbiota analyses

DNA was extracted from mice’ fecal samples using stool DNA extraction kits (Qiagen, Milan, Italy) according to the manufacturer’s instructions and PCR amplification of the V3-V4 hypervariable regions of 16S rRNA genes was performed using universal Illumina adapters: forward primer: 5′TCGTCGGCAGCGTCAGATGTGTATAAGAGACAGCCTACGGGNGGCWGCAG; reverse primer: 5′GTCTCGTGGGCTCGGAGATGTGTATAAGAGACAGGACTACHVGGGTATCTAATCC, as reported in Klindworth et al. [9] and [10]. The amplified region of about 450 bp and in average 12,000 reads per sample was obtained. The mixture of amplicons was sequenced using Illumina MiSeq 2 × 250 v2 platform. Sequences were demultiplexed based on index sequences and FASTQ files were generated. Sequence data were analyzed as already described [6] using an open source program (BION-meta). Briefly, sequences were trimmed at both ends using a cut-off for minimum quality of 95%, and then reads shorter than 350 bp were removed. Consensus reads were clustered based on a minimum seed similarity of 99.5% and then taxonomically aligned to the reference 16S rDNA database (SILVA) using a match minimum of 90%.

### LC–MS analysis of serum metabolomics

Serum proteins were precipitated using acetonitrile. The supernatant was diluted 1:1 in a acetonitrile/milliQ water mixture. Liquid chromatography/Mass spectrometry was used to analyze serum metabolites. The samples were analyzed using a slightly modified version of the acidic protocol (positive and negative ionization) described by Paglia et al. [11]. For quality control, a mixed pooled sample was created by taking a small aliquot from each sample. A targeted approach was used to extract the response of compounds included in a standard list of 97 compounds.

### Data processing

Data were processed using Compound Discoverer 3.0 (ThermoFisher Scientific). In short features (a feature is a peak characterized by one mass and one retention time) were first extracted from the raw data. One compound may give a signal in more than one mass trace (e.g. naturally occurring C13 isotopes, adducts, and fragments) a compound will therefore almost always be represented by more than one feature with the same retention time but different masses. The feature detection is followed by grouping of features belonging to the same compound. This additional information (e.g. isotope pattern) is then used together with the accurate mass to determine the molecular formula. The mass and molecular formula is then used to perform a search in a suitable database using ChemSpider.

### Statistical analysis

In vitro and in vivo results are expressed as means ± SE. Comparisons were made using Student’s t-test. Differences were considered as significant when *P* < 0.05 (*) or *P* < 0.01 (**).

## Results

### Effect of DCA on microbiota profile of PC xenograft mice

**Fig. 1:**
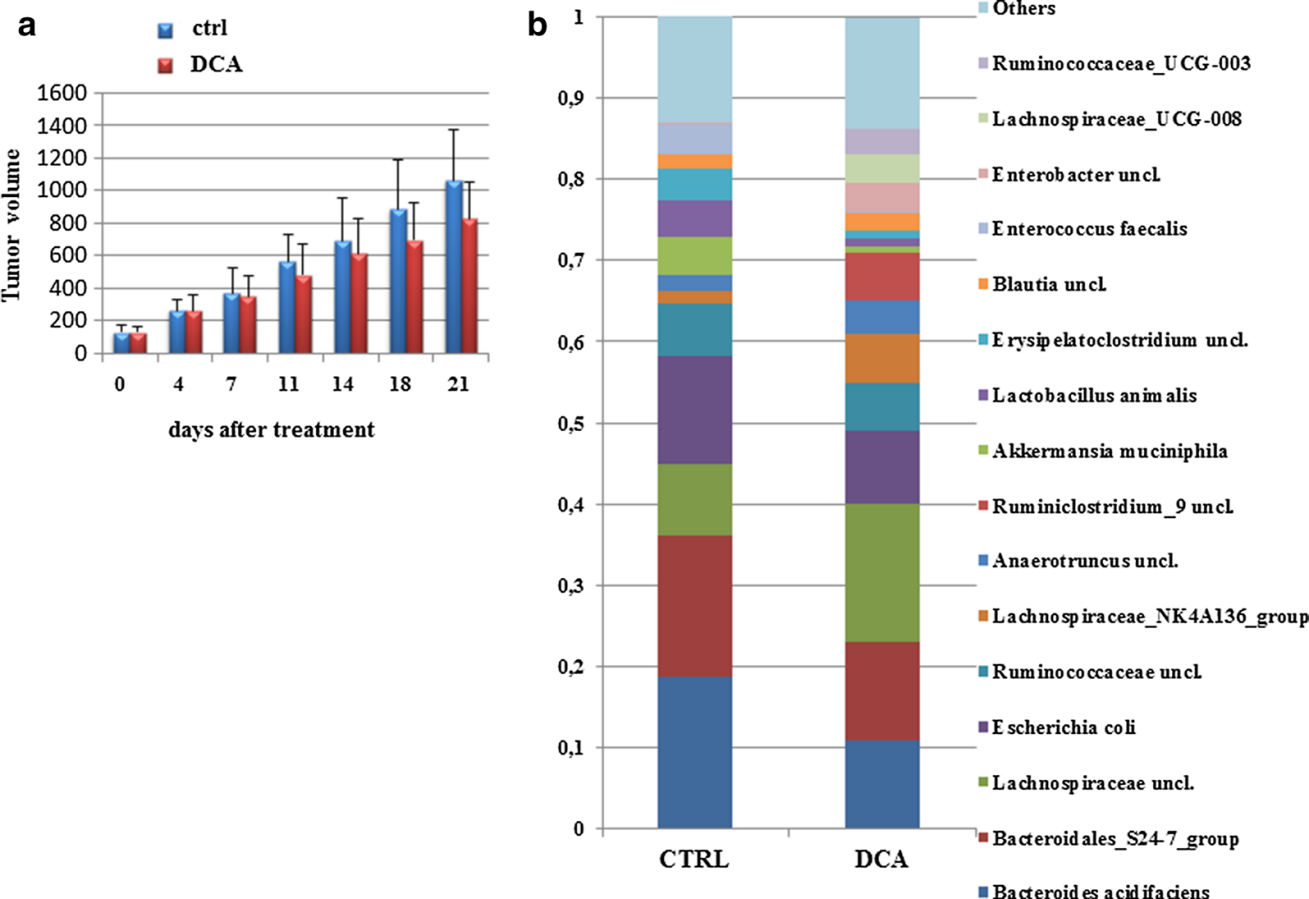
Tumor volume evaluation along the treatment. **a** Results are expressed as means ± S.D. Differences were considered as significant when *p* < 0.05 (*). Mean composition of bacterial taxa in mice fecal samples. **b** The average abundance (average sum of reads in relative scale, %) of the most represented 15 bacterial taxa is shown

Beside the trend in retarding tumor growth without reaching statistical significance (Fig. 1a) as previously published [11], DCA treatment did not have significant effect on the fecal microbiota on the phylum level and the two main phyla were Firmicutes and Bacteroidetes with lower abundance of Proteobacteria and Verrucomicrobia (average abundances: 39, 38, 17 and 5%, and 58, 24, 17 and 1% in control and DCA mice, respectively). However, at the family, genus or species level DCA treatment led to several shifts on the microbiota. DCA treated mice can be characterized by dominance of Lachnospiraceae (32 vs 16% in DCA and control mice, respectively) and Ruminococcaceae (22 vs 10% in DCA and control mice, respectively). More specifically, there were three genera abundant in DCA mice but absent in control group such as Ruminiclostridium 9 (6% from total population), Lachnospiraceae cluster UCG-008 (3.5%) and Ruminococcaceae cluster UCG-003 (3.3%). Less pronounced but significant increase was also observed in bacteria belonging to Anaerotruncus (5 and 2%, respectively) (Fig. 1b). All these changes mentioned above were conversely observed in case of gemcitabine treatment as published previously [12]. The only bacteria observed only in control group were genus Coprococcus 1 although this was low abundant genus (0.3%). Also but not significantly Lactobacillus animalis representing about 4% of the bacterial abundance in controls was only 1% in treated animals. Similarly, bacteria belonging to Aeromonas were more abundant in control group (1.3 vs 0.3%, respectively).

### Metabolomic profile in DCA treated xenograft mice

**Fig. 2:**
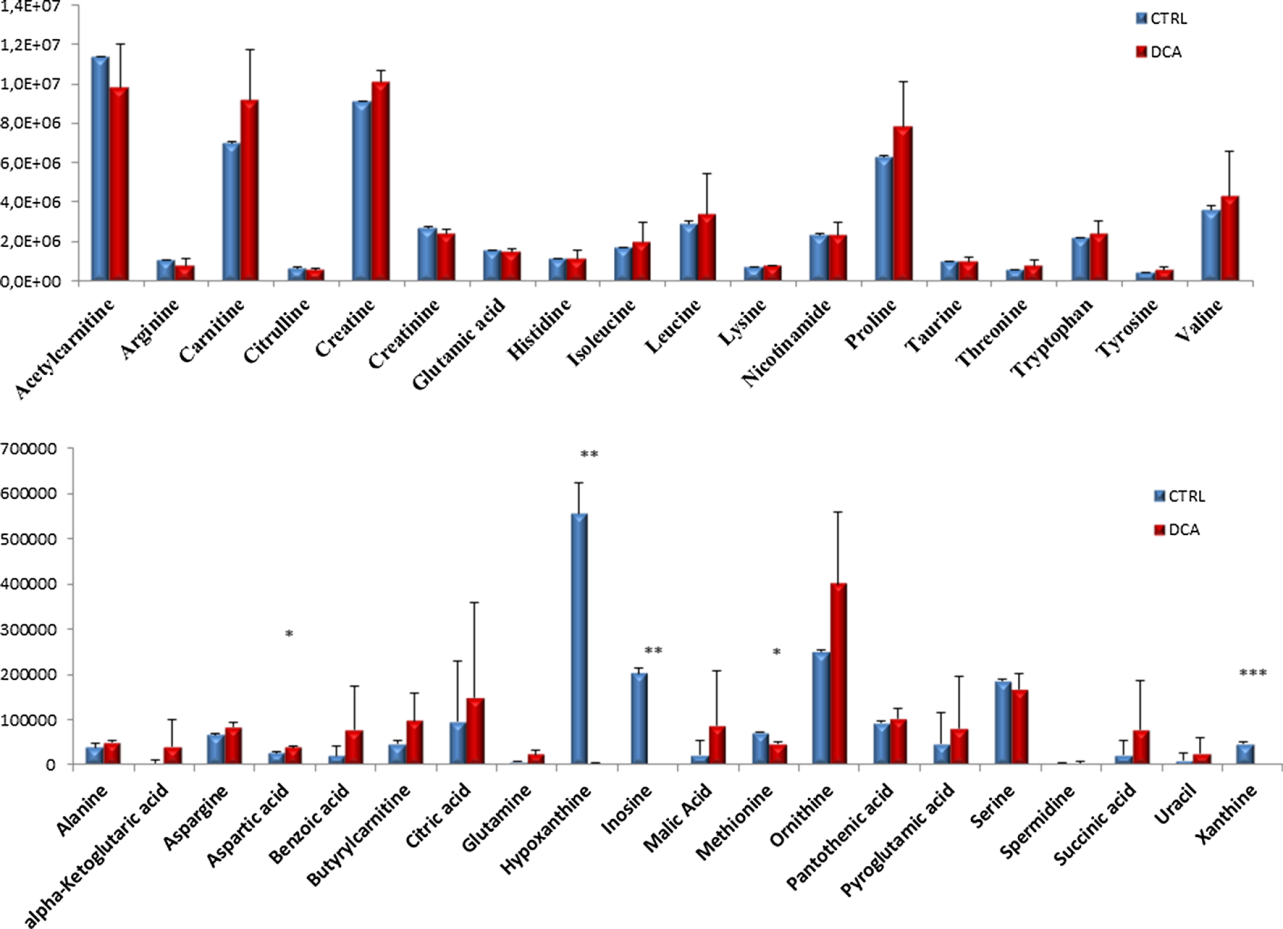
Bar charts representation of the 38 metabolic compounds detected in control and DCA treated mice serum samples. Results are expressed as means ± SD. Differences were considered as significant when *p* < 0.05 (*), *p* < 0.01 (**) or *p* < 0.001 (***)

Serum samples collected from both control and DCA-treated animals were collected to evaluate the metabolomic profile. Using a targeted approach, A total of 38 out of 97 compounds were detected (Fig. 2). Among these compounds, a significant decrease of the purine bases inosine xanthine together with their metabolically-related compound hypoxanthine were observed in the DCA treated group as compared to the control group. Two aminoacids methionine and aspartic acid resulted decreased and increased respectively.

## Discussion

The study of the microbiota has attracted increasing attention during the last decade since it has become clear that the composition and function of this ecological community of commensal, symbiotic and pathogenic microorganisms affect several physiological and pathological processes, including cancer [13]. Although microbiota is relatively stable over time [14] it can be easily modified by a number of environmental factors, such as lifestyle, diet and drugs [15–17]. The current study shows that DCA treatment led to several shifts on the microbiota in particular at family, genus or species level in pancreatic cancer xenograft mice. Above all, DCA treatment significantly increased the relative abundance of *Lachnospiraceae.* Several species from Lachnospiraceae are fiber degrading and butyrate producing bacteria, hence, they might have beneficial effects on cancer inhibition. Butyrate has shown anti-proliferative and pro-apoptotic effects not only in pancreatic cancer cells but also in different cancer cell lines together with anti-angiogenic properties [18, 19]. Moreover, butyrate inhibits pancreatic cancer cells vitro invasion by altering integrin expression [20]. Host metabolic phenotype is impacted by the interaction between bacteria and the host. For this reason we performed a metabolomics targeted analysis of mice serum. Beside the decreased level of inosine, xanthine and hypoxantine that we discussed in our previous work [12] we also observed a decreased level of the essential aminoacid methionine.

Inosine, which is a naturally occurring metabolite of adenosine, possesses anti-inflammatory and immunosuppressive properties and has a protective effect against LPS-induced inflammation [21]. Interestingly, decreased levels of inosine could also be due to the microbiota itself, since it has been reported that Lactobacillus reuteri impacts inosine levels [22]. As for methionine, several studies showed that methionine restriction also inhibits aging-related disease processes in mice and inhibits colon carcinogenesis in rats [23] and in humans, methionine restriction could be achieved through a plant-based diet [24]. In conclusion, these data suggest a role for DCA on tumor environment and more studies are needed in order to understand whether DCA supplementation could be supportive as synergistic approach to enhance the efficacy of existing cancer treatments in pancreatic cancer patients.

## Limitations

The current study reports that DCA administration affects gut microbiota and metabolomic profiles of pancreatic cancer xenograft mice. Future investigations are needed in order to understand whether DCA supplementation could be supportive as synergistic approach to enhance the efficacy of existing cancer treatments in pancreatic cancer patients.

## Data Availability

All data generated and analyzed during this study will be freely available to any scientist wishing to use them for non-commercial purposes upon reasonable request.

## Abbreviations

PC: Pancreatic cancer
DCA: Dichloroacetate
PDK: Pyruvate dehydrogenase kinase
PDH: Pyruvate dehydrogenase
